# Interkingdom Cross-Talk in Times of Stress: *Salmonella* Typhimurium Grown in the Presence of Catecholamines Inhibits Porcine Immune Functionality *in vitro*

**DOI:** 10.3389/fimmu.2020.572056

**Published:** 2020-09-30

**Authors:** Lena Reiske, Sonja S. Schmucker, Julia Steuber, Charlotte Toulouse, Birgit Pfaffinger, Volker Stefanski

**Affiliations:** ^1^Behavioral Physiology of Livestock, Institute of Animal Science, University of Hohenheim, Stuttgart, Germany; ^2^Cellular Microbiology, Institute of Biology, University of Hohenheim, Stuttgart, Germany

**Keywords:** *Salmonella* Typhimurium, catecholamines, adrenaline, adrenochrome, pig, stress, interkingdom signaling, immune function

## Abstract

In stressful situations, catecholamines modulate mammalian immune function, and in addition, they can be sensed by many bacteria. Catecholamine sensing was also found in the zoonotic gut pathogen *Salmonella* Typhimurium, probably contributing to the stress-induced increased risk of salmonellosis. Virulence traits such as proliferation and invasiveness are promoted upon bacterial catecholamine sensing, but it is unknown whether *S.* Typhimurium may also inhibit mammalian immune function in stressful situations. We thus investigated whether supernatants from *S.* Typhimurium grown in the presence of catecholamines modulate porcine mitogen-induced lymphocyte proliferation. Lymphocyte proliferation was reduced by supernatants from catecholamine-exposed *Salmonella* in a dose-dependent manner. We further examined whether adrenaline oxidation to adrenochrome, which is promoted by bacteria, could be responsible for the observed effect, but this molecule either enhanced lymphocyte functionality or had no effect. We could thereby exclude adrenochrome as a potential immunomodulating agent produced by *S.* Typhimurium. This study is the first to demonstrate that bacteria grown in the presence of catecholamine stress hormones alter their growth environment, probably by producing immunomodulating substances, in a way that host immune response is suppressed. These findings add a new dimension to interkingdom signaling and provide novel clues to explain the increased susceptibility of a stressed host to *Salmonella* infection.

## Introduction

In acute stress situations, the mammalian body launches a rapid physiologic response, which enables it to cope with threats imposed on its health. In the course of such a “fight-or-flight” reaction, substantial amounts of stress hormones, particularly adrenaline (ADR) and noradrenaline (NA), can be released from the adrenal gland and at sympathetic nerve endings. These catecholamines (CAs) not only exert effects on blood circulation, respiration, energy metabolism, and many other functions supporting physical exertion (1–3), but also affect the immune system (4, 5). The long-held view of general immunosuppression by stress hormones was increasingly challenged in recent years, as especially CA actions are rather diverse and dose-dependent, including both inhibiting and enhancing actions (5–9). In some organs, such as the spleen or the gut, stress-related CA release can lead to local concentrations of up to 10^–4^ to 10^–3^ M (10, 11), which is much higher than in the blood, where levels are between 10^–9^ and 10^–6^ M (12, 13). This is caused by NA discharge from synaptic vesicles at noradrenergic nerve endings (10, 11, 14). In the gut and other tissues with contact to the external world via epithelial surfaces, CAs can even cross the epithelial border and interact with microorganisms living in those ecological niches (15–18). In the colon, NA can reach a concentration of about 50 ng/g luminal content (14).

In the last two decades, more and more studies in the field of microbial endocrinology emerged, investigating the cross-talk between the endocrine and nervous system of host species and microorganisms inhabiting or invading them. A plethora of microorganisms exist naturally as commensals, e.g., in the gut, oral cavity, and on the skin (19–21). It is therefore no surprise that both parties evolved mechanisms to communicate with each other via mammalian hormones and hormone-like microbial molecules, with mutual benefits supporting symbiosis. However, many pathogens have been proven to sense stressful situations with high CA levels and exploit them by boosting virulence (22, 23). NA can be used by many bacterial species as an iron donor (24, 25) or activate quorum sensing–a bacterial cell-to-cell communication–by directly binding to QseC or QseE (26–28). Elevated ADR and NA concentrations can thus lead to an increased bacterial growth rate (29, 30), motility (26, 29), or attachment to epithelial surfaces (22)–in short, higher chances of infection. This interkingdom signaling works in both directions. Independently of host stress, bacteria produce molecules for interbacterial communication, some of which have a hormone-like side effect on host cells (31). For instance, many Gram-negative bacteria produce substances, which are chemically analogous to eukaryotic lipid hormones and can modulate host immune functions such as neutrophil chemotaxis and lymphocyte proliferation (32–35). Moreover, some quorum-sensing molecules produced by several regular inhabitants of the gastrointestinal tract (GIT) probably act as agonists at adrenergic receptors (ARs) (36).

Regarding this intense cross-talk between kingdoms, it is conceivable that in stressful situations, pathogens not only modulate their own properties but may even actively manipulate immune cells to exploit a weakened host. Upon CA perception, they might react with the release of bacterial hormone-like molecules similar to the aforementioned ones. Furthermore, a microbial alteration of mammalian CAs might lead to the formation of an immunomodulating substance. CAs are vulnerable to oxidation (37), and in the presence of superoxide, the oxidation of ADR to adrenochrome (AC) is promoted (38). A boost of AC formation by superoxide-producing bacteria might cause immunomodulation as it was shown that AC can bind to β-ARs (39), which can be found on most immune cells (40). Indeed, it was demonstrated in *Vibrio cholerae* O395N1 that the bacterial Na^+^-translocating NADH:quinone oxidoreductase (NQR) promoted the oxidation of ADR to AC by superoxide production (41). AC supported the pathogenicity of *V. cholerae* by stimulating its growth even stronger than ADR and in addition exerted immunomodulating effects by inhibiting tumor necrosis factor α (TNF-α) production in a human monocytic cell line (41). It can be hypothesized that *V. cholerae* is not the only gut pathogen capable of this reaction, and the promotion of AC formation may be a strategy also used by other bacteria to manipulate host immune functionality. An interesting candidate to test this hypothesis is the important zoonotic gut pathogen, *Salmonella enterica* ssp. *enterica* serovar Typhimurium (*S.* Typhimurium), which is common in domestic pigs (*Sus scrofa domestica*) and difficult to eradicate. It is known that stress has a negative impact on primary *Salmonella* infection in pigs and also on the recrudescence of asymptomatic latent infections, for example, by transportation to the slaughterhouse (42). The resulting bacterial shedding by slaughter pigs leads to increased carcass contamination and thus intensifies the risk of food-borne transmission to humans (43). However, despite the importance of this bacterial infection both from a veterinary and a medical point of view, the underlying mechanisms of these observations are still not sufficiently resolved. Because an enhanced motility and growth rate upon CA sensing have also been found in *Salmonella* (16, 26), studying interkingdom signaling is a promising approach to better explain the promotion of salmonellosis by stress.

The aim of the present study was thus to investigate whether *S.* Typhimurium grown in the presence of CAs has the potential to hamper porcine immune functionality. We examined the effects of supernatants from *S.* Typhimurium cultures exposed to NA or ADR on lymphocyte proliferation and demonstrated an inhibitory effect. Furthermore, we investigated whether AC is the causative agent of this inhibition.

## Materials and Methods

### Animals and Sampling

To obtain blood for *in vitro* studies without stress hormone release during the sampling procedure, 37 castrated male pigs (German Landrace × Pietrain, age 7 months) with indwelling vein catheters were used in total. At least 14 days before the beginning of blood sampling, *Vena cephalica* cannulation was performed under generalized anesthesia. Surgery was performed as previously described (44) with few modifications (45). The barrows were housed individually in pens (5.4 m^2^) with visual and tactile contact to their conspecifics. Pens were littered with dust-free wood shavings and cleaned every day after feeding. Light was on from 06:30 until 20:30. Pigs were fed hay *ad libitum* and concentrate (1.5 kg/meal, ME 12 MJ/kg) twice a day in the morning at 07:30 and in the afternoon at 15:00. To ensure blood sampling without disturbance of the animals, pigs were thoroughly habituated to human handling. Catheters were rinsed with heparinized saline (115 IU/mL; B. Braun Melsungen AG, Melsungen, Germany) every day during feeding in the morning. For blood collection via the catheters, 5 mL of blood was drawn and discarded before 10 mL blood per animal was collected into lithium heparin tubes (Sarstedt, Nümbrecht, Germany). Separation of peripheral blood mononuclear cells (PBMCs) from whole blood was performed with Leucosep^TM^ Centrifuge Tubes (Greiner Bio-One, Frickenhausen, Germany) using Biocoll with a density of 1.077 g/mL (Biochrom, Berlin, Germany) as previously described (6). In brief, PBMCs were separated by a density gradient, and after two washing steps; cells were suspended in RPMI 1640 supplemented with 10% fetal calf serum (FCS) and 50 μg/mL gentamycin (all Biochrom). Afterward, cell concentration was determined with a Z2 Coulter Counter (Beckman Coulter, Krefeld, Germany).

### Preparation of Bacterial Supernatants

To acquire supernatants from bacteria grown *in vitro* in presence and absence of 0.1 mM ADR, 0.1 mM NA, or 0.02 mM AC (Sigma-Aldrich, Taufkirchen, Germany), *S. enterica* serovar *Typhimurium* Zoosaloral his^–155^/ade^–4^ (*S.* Typhimurium; DSM-No: 11320), auxotroph for histidine and adenine was chosen. *S.* Typhimurium was first allowed to grow on LB agar overnight at 37°C [1% (wt/vol) tryptone, 0.5% (wt/vol) yeast extract, 1% (wt/vol) NaCl, and 1.5% (wt/vol) bacto agar]. A single colony was used to inoculate 25 mL of LB medium [1% (wt/vol) tryptone, 0.5% (wt/vol) yeast extract, 1% (wt/vol) NaCl]. After incubation overnight at 37°C and 180 rpm shaking (Infors HT Ecotron), *S.* Typhimurium cells were harvested by centrifugation (3 min, 10,000 × *g*), washed, and resuspended in heat-treated serum-SAPI cultivation medium (29) to obtain an optical cell density at 600 nm of 2 (Diode Array HP 8462A, Hewlett Packard, Palo Alto, CA, United States). Heat-treated serum-SAPI cultivation medium contains SAPI solution [6.25 mM NH_4_NO_3_, 1.84 mM KH_2_PO_4_, 3.35 mM KCl, autoclaved; 1.01 mM MgSO_4_, 2.77 mM glucose, 10 mM HEPES pH 7.5 sterile filtered (0.22 μm)], 30% (vol/vol) FCS (Sigma-Aldrich), which was heat inactivated at 55°C for 20 min prior to use and supplementation of 0.12 mM adenine monohydrochloride and 0.13 mM L-histidine. Serum-SAPI was used as it is the medium of choice for analysis of CA effects on bacteria (15, 29, 30). Cultivation medium was inoculated with the cell suspension to obtain an OD_600_ of 0.01. To triplicates of 20 mL inoculated serum-SAPI either 10^–4^ M ADR, 10^–4^ M NA, or 2 × 10^–5^ M AC (Sigma-Aldrich), or no further compound was added and incubated at 37°C and shaking (180 rpm). As control, cultivation medium without bacterial cells and without CAs or AC was also incubated under the same conditions. After 8 h of growth, when cells were in the exponential growth phase, cells were harvested by centrifugation (15 min, 7,000 rpm) and the supernatant was sterile filtered (0.22 μm), frozen in liquid nitrogen, and stored at -80°C. Cells were harvested for collection of supernatants at OD600 = 0.34 (no addition), 0.47 (ADR), 0.49 (NA), and 0.36 (AC).

### Determination of CA Contents in Bacterial Supernatants via High-Performance Liquid Chromatography

High-performance liquid chromatography (HPLC) with electrochemical detection was conducted to determine the concentration of CAs in bacterial supernatants grown in the presence of NA or ADR. The HPLC system (ISO-3100BM, Thermo Fisher Scientific) was connected to an electrochemical detector [Coulochem III, conditioning cell (model 50210A), analytical cell (model 5011A), Thermo Fisher Scientific]. The potentials of the cells were set at 300, 50, and -250 mV. The system was equipped with the column Reprosil Pur 120 C18-AQ (4.6 × 75 mm) (A. Maisch, Ammerbuch, Germany). Cat-A-Phase II was used as the mobile phase, with a flow rate of 1.1 mL/min. The sample preparation with alumina extraction were adapted from the method first described by Anton and Sayre (46). Bacterial supernatants were diluted (1:10,000 and 1:20,000) to be in the range of the applied calibration curve. In brief, 1 mL of sample and 500 pg of an internal standard (dihydroxybenzylamine; Thermo Fisher Scientific, Darmstadt, Germany) were added to extraction tubes containing 20 mg aluminum oxide previously activated with 600 μL 2 M Tris/EDTA buffer (pH 8.7). Samples were thoroughly mixed in an overhead shaker for 10 min and centrifuged at 1,000 × *g* for 1 min (4°C). Samples were washed three times with 1 mL of 16.5 mM Tris/EDTA buffer (pH 8.1), followed by centrifugation. The CAs were eluted by addition of 120 μL eluting solution (Recipe, Munich, Germany), short mixing, and centrifugation at 1,000 × *g* for 1 min (4°C). Aliquots of 50 μL were injected into the HPLC system. The internal standard method using peak areas was applied to evaluate the concentration of the samples.

### Lymphocyte Proliferation Assay

For investigation of lymphocyte proliferative capacity, a mitogen-induced lymphocyte proliferation assay was performed as previously described (47). In short, PBMCs were seeded into 96-well round-bottom cell culture plates (Neolab, Heidelberg, Germany) with 1.5 × 10^5^ cells/well and either stimulated with 5 μg/mL concanavalin A (ConA) or 5 μg/mL pokeweed mitogen (PWM) (both Sigma-Aldrich) or left without stimulation. Subsequently, supernatants from the differently treated *S.* Typhimurium cultures were added in concentrations of either 5, 10, or 15% of the total cell culture volume. To guarantee similar growth conditions throughout the wells, pure serum-SAPI was applied to control wells as well as for volume compensation, resulting in 15% serum–SAPI–based additive in every well. Each treatment was done in triplicates. Cells were incubated at 39°C, and 5% CO_2_ for 48 h before 0.25 μCi ^3^H-thymidine/well (PerkinElmer, Rodgau, Germany) was added, followed by a further incubation for 24 h. PBMCs were harvested using glass fiber filters (Sigma-Aldrich), and the incorporated radioactivity was measured by a liquid scintillation analyzer (PerkinElmer). For each treatment, the mean of counts per minute (cpm) was calculated, and the mean cpm of the unstimulated control was subtracted to gain Δcpm.

HPLC analysis of the *Salmonella* supernatants showed that substantial amounts of CAs were still present in CA-treated cultures. We thus performed an additional experiment to ensure that probable bacterial effects were not in fact caused by CAs or by mere synergistic effects of bacterial products and CAs. Therefore, previously frozen PBMCs of three animals were thawed and seeded with 1.5 × 10^5^ cells/well in 96-well round-bottom cell culture plates in RPMI 1640 supplemented with 10% FCS and 50 μg/mL gentamycin. Cells were incubated at 39°C and 5% CO_2_ as described for the first experiment after adding one of the following treatments: PBMCs were either left unstimulated after addition of 15% serum-SAPI medium or stimulated with 5 μg/mL ConA. Stimulated cells were supplemented with one of the following additives: 15% serum-SAPI alone, 15% serum-SAPI and 10^–5^ M NA, 15% serum-SAPI and 10^–5^ M ADR, 15% supernatants from *S.* Typhimurium grown without hormone, 15% supernatants from *S.* Typhimurium grown in the presence of 10^–4^ M NA or 10^–4^ M ADR, or 15% supernatants from *S.* Typhimurium grown without hormone with retrospective addition of 10^–5^ M NA or 10^–5^ M ADR.

In a third experiment, lymphocyte proliferation was assessed again as described previously but with addition of AC (Sigma–Aldrich). As the effective concentration (and the amount of presumed ADR oxidation in *Salmonella* cultures) was unknown, we investigated a wide range of concentrations (10^–10^ to 10^–5^ M). After addition of AC and stimulation with 5 μg/mL ConA or 5 μg/mL PWM, cells were incubated, and proliferation was determined as described above.

### Statistical Analysis

For statistical analysis, we used the software SAS, version 9.4 (SAS institute Inc., Cary, NC, United States), applying the MIXED procedure. Degrees of freedom were determined with the Kenward–Roger method (48); normal distribution and variance homogeneity were confirmed visually by normal probability plots and plots of residuals versus fitted values (49). For estimation of variance components, we used the restricted maximum likelihood method. The models included the factors “treatment” and “trial,” as well as their interaction as fixed effects and “sampling day” and “sampling day × treatment” as random effects. To take into account the individual level of the pigs, “animal” was included as a repeated effect. If data were not normally distributed, logarithmic or square root transformation was performed to attain normality. The results are presented as least square (ls)-means + standard error of the mean (SEM). Statistically significant differences were determined by Fisher’s least significant difference test. Significance limits were set as follows: ^∗^*p* < 0.05, ^∗∗^*p* < 0.01, ^∗∗∗^*p* < 0.001, and ^*t*^*p* < 0.1 (tendency).

## Results

### Supernatants From CA-Treated *S.* Typhimurium Cultures Inhibit Lymphocyte Proliferation

**FIGURE 1:**
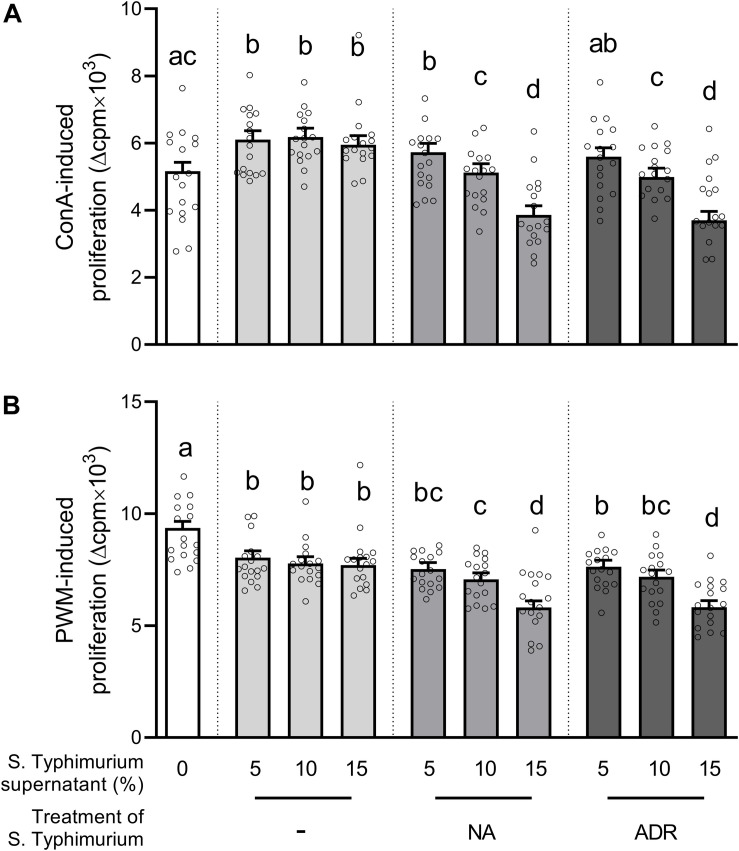
Lymphocyte proliferation after stimulation with either **(A)** concanavalin A (ConA) or **(B)** pokeweed mitogen (PWM), as well as addition of either serum-SAPI medium (white) or supernatants from *Salmonella* Typhimurium cultures grown for 8 h at 37°C without hormones (light gray) or in the presence of 10^–4^ M noradrenaline (NA; medium gray) or 10^–4^ M adrenaline (ADR; dark gray). Supernatants were added in concentrations of either 5%, 10%, or 15% of the cell culture volume as indicated on the *x* axis. Treatments that are statistically significant from each other are indicated by different letters on top of their bars, whereas bars that share a common letter do not differ significantly. Data are presented as ls-means + SEM (bars) and single values of each animal (circles), *n* = 16.

We first evaluated the effects of supernatants from *S.* Typhimurium cultures on lymphocyte proliferation. Compared to the media control, the addition of supernatants from hormone-free *Salmonella* cultures enhanced ConA-induced lymphocyte proliferation (Figure 1A). In comparison to supernatants from hormone-free *Salmonella* cultures, lymphocyte proliferation was reduced significantly if 10% or 15% of supernatants from *Salmonella* grown in the presence of ADR or NA were added and already tended to be lower (*p* = 0.053) if 5% of supernatants from *Salmonella* grown in the presence of ADR were added. In PWM-stimulated PBMCs, already the addition of supernatants from hormone-free *Salmonella* cultures reduced proliferation compared to the media control (Figure 1B). But similar to ConA-stimulated cells, addition of 10% or 15% of supernatants from *Salmonella* grown in the presence of NA further reduced proliferation significantly. The addition of supernatants from *Salmonella* grown in the presence of ADR caused a less pronounced suppression of PWM-stimulated cells with a significant effect if 15% and a tendency (*p* = 0.058) if 10% were added.

### Suppression of Lymphocyte Function Is Not Due to CA Action

Because CAs themselves are well-described to modulate immune cell functionality, we determined whether CAs were still present in *S.* Typhimurium cultures incubated for 8 h in the presence of either NA or ADR by HPLC analysis. Thereby, an ADR concentration of 19.67 μg/mL (1.07 × 10^–4^ M) was found, representing the same level as applied at the start of incubation (1 × 10^–4^ M). NA showed a slight decrease compared to the initial concentration of 1 × 10^–4^ M, but was still present in the supernatants at a concentration of 8.08 μg/mL (4.8 × 10^–5^ M). Thus, to verify that probable bacterial effects were not “ordinary” immunomodulating effects of CAs or caused by mere synergistic effects of bacterial products and CAs, we tested the effects of simultaneous addition of supernatant from *S.* Typhimurium grown without hormones and either NA or ADR in the same range as found within the culture supernatants tested in the initial experiment (cf. Figure 1).

**FIGURE 2:**
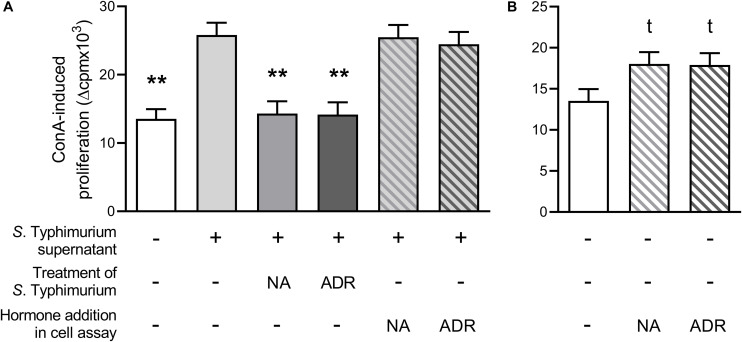
Lymphocyte proliferation after stimulation with 5 μg/mL concanavalin A and upon addition of 15% serum-SAPI (white), 15% supernatants from *Salmonella* Typhimurium cultures grown without hormones (light gray) or grown with either 10^–4^ M noradrenaline (NA; middle gray) or 10^–4^ M adrenaline (ADR; dark gray) for 8 h at 37°C, or addition of 15% supernatants from *S.* Typhimurium cultures grown without hormones simultaneous to catecholamine addition [10^–5^ M NA (light gray hatched in middle gray) or 10^–5^ M ADR (light gray hatched in dark gray)] **(A)**; or upon addition of 15% serum-SAPI without further additives (white) or additional supplementation with 10^–5^ M NA (white hatched in middle gray) or 10^–5^ M ADR (white hatched in dark gray) **(B)**. Data are presented as ls-means + SEM, *n* = 3. Asterisks and t in superscript indicate significant differences and tendencies compared to supernatants from hormone-free *Salmonella* culture **(A)** or the hormone-free control **(B)**, respectively.

As seen in Figure 2A, ConA-induced lymphocyte proliferation was significantly lower if supernatants from *Salmonella* grown in the presence of NA or ADR were added compared to supernatants from hormone-free *Salmonella* culture. Thus, the results presented above (cf. Figure 1) could be confirmed. Notably, in contrast to this effect, no suppression occurred on ConA-induced lymphocyte proliferation if supernatants from hormone-free *Salmonella* cultures were added simultaneously with ADR or NA (Figure 2A). Opposite to the effect of supernatants from *Salmonella* grown in the presence of NA or ADR, proliferation was slightly increased if cells were treated with NA (*p* = 0.073) or ADR (*p* = 0.068) alone (Figure 2B).

### The ADR Oxidation Product AC Is Not the Active Inhibitory Agent in Supernatants From CA-Treated *Salmonella* Cultures

**FIGURE 3:**
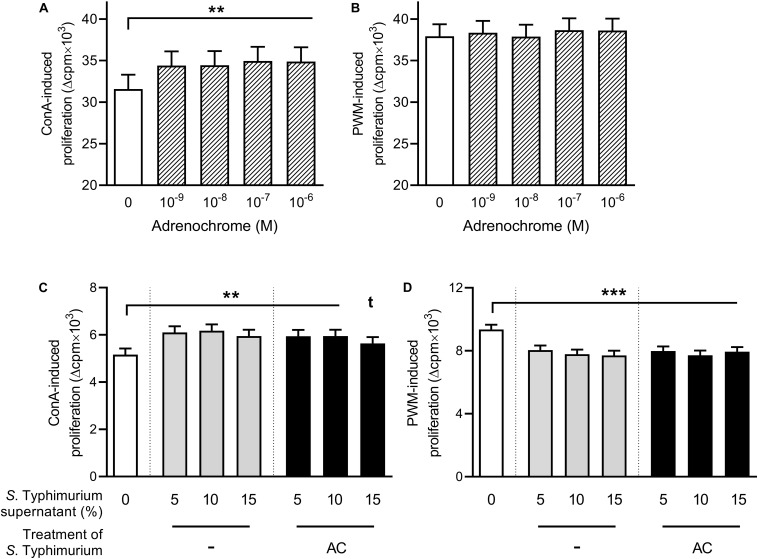
Lymphocyte proliferation upon addition of adrenochrome **(A,B)** or serum-SAPI medium or supernatants from *Salmonella* Typhimurium cultures grown without additive or in the presence of 2 × 10^–5^ M adrenochrome for 8 h at 37°C **(C,D)**, and stimulation with either 5 μg/mL concanavalin A [ConA; **(A,C)**] or 5 μg/mL pokeweed mitogen [PWM; **(B,D)**]. Significant differences are marked by asterisks, tendencies are indicated by a *t* in superscript. Data are presented as ls-means + SEM, *n* = 19 **(A,B)**, *n* = 16 **(C,D)**.

To assess whether the oxidation of CAs by *Salmonella* might cause the observed suppressive effect of supernatants from CA-treated bacterial cultures, we performed the lymphocyte proliferation assay under the same conditions as in the first experiment (cf. Figure 1) but added AC instead of bacterial supernatants (Figures 3A,B). If PBMCs were stimulated with ConA, all tested concentrations led to an enhancement of proliferation compared to the AC-free control (Figure 3A), whereas no effect was observed upon stimulation with PWM (Figure 3B).

Because AC can also have a direct effect on bacteria, like in *V. cholerae* (41), we assumed that its effect on PBMCs might possibly be mediated indirectly, by modulating the behavior of *S.* Typhimurium upon sensing. In addition to the treatment of *Salmonella* cultures with NA or ADR, we thus also cultured *S.* Typhimurium with 2 × 10^–5^ M AC for 8 h at 37°C before centrifugation and microfiltration. If supernatant from these cultures was added to ConA-stimulated PBMCs, proliferation was enhanced compared to the serum–SAPI–control but not significantly different from the proliferation upon addition of supernatants from hormone-free *Salmonella* cultures (Figure 3C). If PWM was used, proliferation was lower than upon serum-SAPI addition and on the same level as with the supernatant from hormone-free *Salmonella* cultures (Figure 3D).

## Discussion

The results of the present study indicate a close host–pathogen cross-talk in situations with elevated stress hormone levels in pigs. Based on pioneering work demonstrating the ability of many bacteria to increase pathogenicity in response to CAs (23, 50), we here show that interkingdom signaling also works the other way. Our data indicate that there is a direct action of CA-treated bacteria on host immune cells. Lymphocytes treated with cell-free supernatants from *S.* Typhimurium grown in the presence of ADR or NA showed a decreased proliferation, which is probably not the only hampered immune function. Future studies should investigate further important immune functions such as the production of pro-inflammatory cytokines, which are also involved in *Salmonella* control (51).

We demonstrate that the inhibition of lymphocyte proliferation does not simply reflect an immunomodulating effect of CAs, as retrospective addition of ADR or NA in combination with supernatant of non-treated *S.* Typhimurium did not inhibit mitogen-induced proliferation of porcine immune cells. This is also supported by our previous study, showing that under the same cell culture conditions, the sole addition of ADR or NA led to an increased lymphocyte proliferation instead of its reduction (6). This implies that the proposed immunosuppressive substance produced by CA-treated *S.* Typhimurium must be very potent if it even diminishes the enhancing effect of the CAs that were still present in the supernatants.

To the best of our knowledge, this is the first study to report that bacteria grown in the presence of stress hormones alter their growth environment—probably by producing immunomodulating substances—in a way that host immune response is impaired.

Based on own previous studies, AC was a promising candidate for the observed immunosuppression by *S.* Typhimurium. These experiments demonstrated that AC was formed during bacterial culture of *V. cholerae* (29, 41) upon ADR addition, and AC treatment of the human monocytic cell line THP-1 caused a hampered TNF-α production (41). Also, it is already known that AC can bind to ARs (39), which are present on all immune cells (4). We thus investigated whether this oxidation product of ADR may be responsible for the observed effects on porcine primary immune cells. However, AC either added directly to porcine lymphocytes or added to *S.* Typhimurium cultures did not decrease porcine lymphocyte functionality but instead had no effect or even increased it. Based on these results, it can be ruled out that AC is the immunomodulating substance responsible for the observed inhibition. Thus, *S.* Typhimurium must have produced different signaling molecule(s). At this point, it can only be speculated as to what substance might be responsible for the findings by comparing the demonstrated effects with those attributed to already identified molecules that are produced by *S.* Typhimurium or other bacteria.

It was shown that NA triggers the release of autoinducers (AIs) in many Gram-negative bacteria including *Salmonella* (16). This group of quorum-sensing molecules not only enhances the growth and virulence of the bacteria themselves but may also influence the host immune system. The most prominently mentioned and potentially immunomodulatory AI in the literature is AI-3, which is also produced by *S.* Typhimurium (36, 52). Although the exact structure still remains unknown, it has an aminated aromatic compound and seems to have a high similarity to CAs because it can be blocked by α- and β-adrenergic antagonists (53–55), and both NA and AI-3 can bind to QseC (27). It is thus likely that AI-3 can bind to mammalian ARs. However, we have previously shown by *in vitro* culture with CAs that AR binding leads to increased proliferation of porcine PBMCs, contrary to the effects of supernatants from ADR- or NA-treated *Salmonella* presented here (6). Also, an α-adrenergic action of AI-3 is unlikely as binding to these receptors generally causes an enhanced immune functionality (4, 9). Nevertheless, it cannot be precluded at this point that AI-3 might specifically bind to β_2_-ARs in mammalian immune cells, which are mostly immunosuppressive (56).

There is a second important AI molecule produced by *S.* Typhimurium in the exponential growth phase, named AI-2 (57). It plays a role in invasion and intracellular survival in macrophages (58, 59), but indications for a direct modulation of host immune cells have not been found so far. Whether this is a candidate for immunosuppression by *Salmonella* in a stressed host may be subject of future studies.

Another interesting class of bacterial hormone-like molecules is the lipophilic acyl homoserine lactones (AHLs). They are chemically analogous to eukaryotic lipid hormones and can either impair or exacerbate immune functions, depending on their concentration. It has even been shown that they have the ability to inhibit lymphocyte proliferation and TNF-α production in macrophages and T_*H*_ cells (32, 60, 61). Although this very much resembles the findings of the present study, an AHL production was so far not described in *Salmonella* species (62).

Also, it was shown that *S.* Typhimurium can deacylate the lipid A portion of their lipopolysaccharide, which results in a lower activation of Toll-like receptor 4 on antigen-presenting cells. As a consequence, the immune-activating intracellular nuclear factor κB signaling, as well as the release of pro-inflammatory cytokines, is hampered (63). It is conceivable that the effects observed in the present study may at least partly be caused by an activation of this mechanism upon CA sensing of the bacteria.

Conclusively, this study added further novel clues to explain the increased susceptibility of a stressed host to infection. It has been shown earlier that stress has a negative impact on *Salmonella* recrudescence in pigs by increasing intracellular *Salmonella* proliferation in macrophages (64). A direct effect on invasiveness and intracellular survival rate of *S.* Typhimurium by binding of NA to the histidine kinase QseC was demonstrated in another study in mice (65). *S.* Typhimurium infection in calves was also aggravated by an increase of bacterial proliferation by NA, probably through acting as an iron donor for the bacteria (66). The present work shows for the first time that bacteria grown under the influence of NA or ADR are even able to hamper mammalian lymphocyte functionality. Thus, valuable information is added to the phenomenon of increased *Salmonella* susceptibility of stressed pigs. Pigs represent an important meat-producing agricultural species and are relevant carriers of the widely distributed zoonotic agent *S.* Typhimurium (67). At the same time, pigs are an excellent model for human salmonellosis because porcine nutritional physiology and gut anatomy as well as the immune system are very similar to that of humans (68–71). Upon this basic study, it is thus possible to make presumptions about effects of stress on the risk of salmonellosis in humans, i.e., increased risk of infection due to immunosuppression by CA-primed bacteria, while at the same time gaining knowledge about porcine immunology that may have impacts on pig husbandry and food hygiene at the slaughterhouse.

## Data Availability Statement

The raw data supporting the conclusion of this article will be made available by the authors, without undue reservation.

## Ethics Statement

The animal study was reviewed and approved by the Regierungspräsidium Stuttgart.

## Author Contributions

VS and JS conceived and designed the study. VS, JS, SS, CT, and LR designed the experiments. CT produced bacterial supernatants. BP conducted the CA analyses. LR performed and SSS supervised the immunological experiments. LR analyzed and interpreted the data, and wrote the original draft of the manuscript. VS, JS, SS, CT, and BP contributed to the manuscript preparation. All authors have read and agreed to the published version of the manuscript.

## Conflict of Interest

The authors declare that the research was conducted in the absence of any commercial or financial relationships that could be construed as a potential conflict of interest.
